# Case report: Inferior valved for non-valved glaucoma drainage device exchange for glaucoma control and cosmesis

**DOI:** 10.3389/fopht.2024.1361898

**Published:** 2024-05-31

**Authors:** Abdullah M. Khan, Maram E. A. Abdalla Elsayed, Rizwan Malik

**Affiliations:** ^1^ Division of Pediatric Ophthalmology, King Khaled Eye Specialist Hospital, Riyadh, Saudi Arabia; ^2^ Department of Ophthalmology, Jeddah Eye Hospital, Jeddah, Saudi Arabia; ^3^ Department of Surgery, Sheikh Khalifa Medical City, Abu Dhabi, United Arab Emirates

**Keywords:** glaucoma drainage device, device encapsulation, cosmesis, device exchange, glaucoma surgery

## Abstract

**Introduction:**

While the exchange of a superior valved glaucoma drainage device (GDD) for a non-valved GDD has been reported for achieving glaucoma control, inferior GDD exchange for improving the cosmetic appearance of the eyes due to poor appearance caused by encapsulated GDDs has not been previously documented. Here, we report on two patients with inferior valved GDDs who underwent an exchange for non-valved devices for glaucoma control and cosmetic improvement.

**Case description:**

We report on the case of a 23-year-old gentleman and that of an 8-year-old girl, both of whom had inferior valved GDDs with uncontrolled intraocular pressure and unsightly appearance due to encapsulated GDD plates within the palpebral aperture. Both patients were unhappy about the appearance of their eyes. In each case, improvements in both glaucoma control and cosmesis were achieved by exchanging the valved GDDs for non-valved ones.

**Conclusion:**

Exchanging a valved for a non-valved GDD might help improve the cosmetic appearance of the eyes, in addition to providing glaucoma control.

## Introduction

We describe two patients with inferior valved glaucoma drainage devices (GDDs) who had poor intraocular pressure (IOP) control due to the encapsulation of their GDDs and who were dissatisfied with the unsightly appearance of the devices. These patients subsequently underwent a valved/non-valved GDD exchange both for glaucoma control and cosmesis. While a valved/non-valved GDD exchange has been reported for achieving IOP control (1), this is the first report to document the additional benefit of improved cosmesis in such patients.

## Case description 1

**Figure 1:**
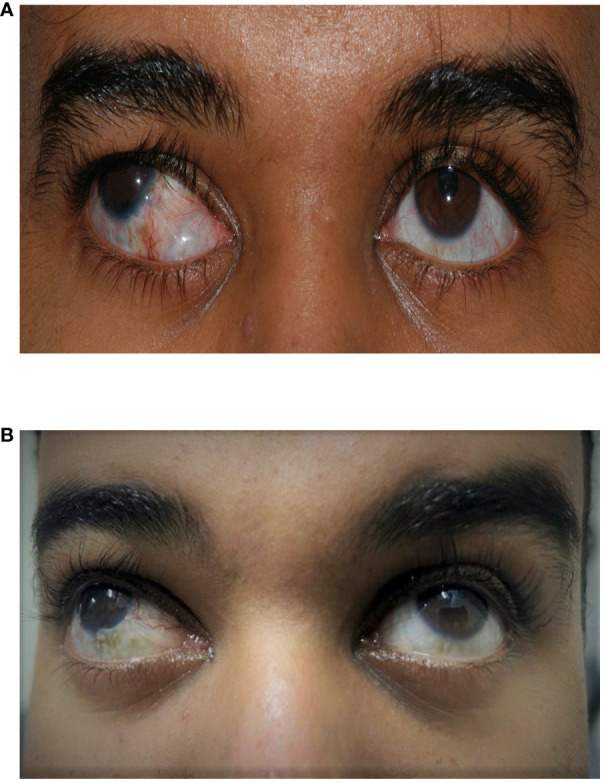
A 23-year-old man who underwent a right inferior Ahmed valve/non-valved exchange. **(A)** Preoperative image showing an inferior encapsulated Ahmed valve. **(B)** Postoperative image showing an improved appearance after exchange for a non-valved glaucoma drainage device (GDD).

A 23-year-old man with a diagnosis of primary congenital glaucoma presented with uncontrolled glaucoma in the right eye. He had previously undergone multiple glaucoma surgeries in the following order: bilateral trabeculotomy, bilateral combined trabeculotomy and trabeculectomy, Ahmed glaucoma valve (AGV) in the superior temporal quadrant in the right eye at the age of 1 year, and then another AGV in the inferior nasal quadrant in the right eye at the age of 15 years. The patient was extremely dissatisfied with the cosmetic appearance due to fibrosis and encapsulation of the inferonasal AGV plate that was visible in the primary gaze (**Figure 1A**). His best corrected visual acuity was 20/400, and the IOP was 30 mmHg despite using four topical anti-glaucoma medications in the right eye. The patient had sensory exotropia of 50 prism dioptres. He had 0.9 cupping of the right optic nerve and advanced visual field loss. His left eye had a best corrected visual acuity of 20/40, and the IOP was controlled after combined trabeculotomy and trabeculectomy at the age of 1 year. He was using two topical anti-glaucoma medications, and the left optic nerve appeared relatively healthy (with a cup/disc ratio of 0.4).

The surgical options considered for his right eye were a second non-valved GDD, cyclodestruction, or an exchange of his inferior AGV for a non-valved GDD. This last option was undertaken as it was expected to improve the poor cosmesis resulting from the encapsulated AGV. The patient was agreeable with this surgical option. The exchange surgery was undertaken using a previously described technique (1). Briefly, after the conjunctival peritomy, both the inferior rectus and the medial rectus were identified to avoid damage during dissection. The AGV plate was then retrieved while leaving the tube in place inside the anterior chamber (AC). Viscoelastic was injected to maintain the AC. An Aurolab aqueous drainage device [Aurolab aqueous drainage implant (AADI); Aurolab, Madurai, India] was intubated with a ripcord of 4–0 nylon and ligated with 7–0 vicryl and one 9–0 nylon sutures to prevent hypotony post-surgery. Occlusion of the tube was assessed by flushing the tube with a balanced salt solution (BSS). Thereafter, the AADI plate was placed under the muscle and fixed to the sclera using two 9–0 nylon sutures. Subsequently, the old AGV tube was removed and the new AADI tube was inserted through the same sclerotomy. The tube was fixed to the sclera with a 9–0 nylon mattress suture. A wick fenestration was made with a 9–0 vicryl suture to help achieve early IOP control (2). The tube was then covered with a lamellar corneal patch graft using four 10–0 nylon sutures. The ripcord was placed in inferior temp fornix (for future retrieval in the clinic). The conjunctiva was closed with continuous 9–0 vicryl sutures and the viscoelastic removed at the end of the surgery.

Postoperatively, the patient had limited early follow-up with his primary physician and needed to be seen by another ophthalmologist in his city during the coronavirus disease 2019 (COVID-19) pandemic. The ripcord was removed 4 months later in the clinic. Since then, the IOP has remained under 18 mmHg on topical medication. The patient’s optic disc and visual field remained stable following the exchange. The patient was very happy with the results of the surgery and the appearance of his eye following the exchange (**Figure 1B**).

## Case description 2

**Figure 2:**
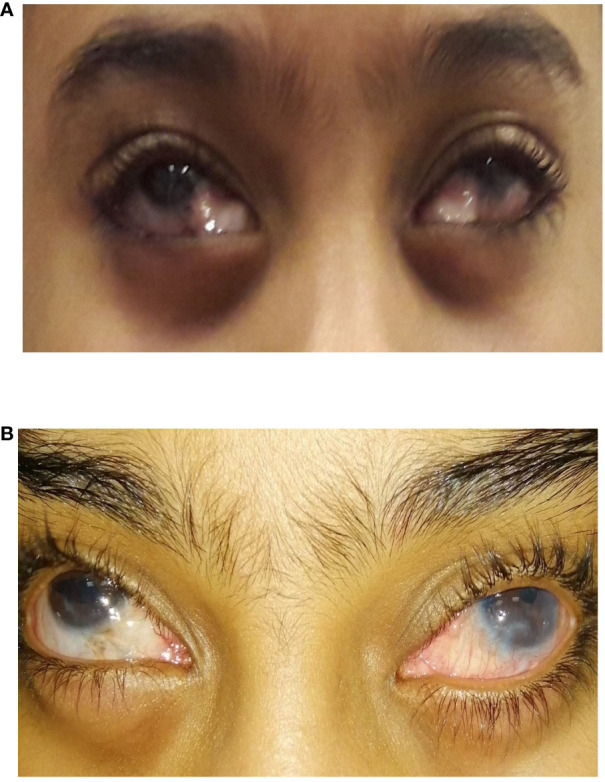
An 8-year-old girl who underwent bilateral inferior Ahmed valve/non-valved exchange. **(A)** Preoperative image showing inferior encapsulated Ahmed valves. **(B)** Postoperative image showing an improved appearance after exchange for non-valved glaucoma drainage devices (GDDs).

An 8-year-old girl presented with uncontrolled glaucoma in both eyes and encapsulated inferior AGVs. She had previously received multiple previous surgeries in both eyes. To summarize, in the right eye, she had one trabeculotomy, four AGV implantations, and three revisions of the capsule of the existing AGVs. In the left eye, she received two trabeculotomies, four AGV implantations, three revisions or dissection of the capsule of the existing AGVs, and one cyclodiode laser ablation. She had multiple tube exposures in both eyes previously. The family and the patient started to notice the fullness and unacceptable cosmesis of both eyes (see **Figure 2A**). The IOP levels in the right and left eyes were 37 and 38 mmHg, respectively, with maximum topical medication in both eyes. Her best correct visual acuity was 20/100 in the right eye and 20/160 in the left eye, with optic disc cupping of 0.8 and 0.9 in the right and left eyes, respectively, and advanced visual field loss in both eyes.

Surgical options were limited due to extensive conjunctival scarring in all quadrants in both eyes. Cyclodiode photocoagulation and an exchange of her inferior AGVs for non-valved GDDs were considered. As the latter option had the potential to improve the cosmetic appearance of her eyes, the patient and her parents opted for this option. The decision to perform an exchange of the AGV implant for a Baerveldt implant for the left eye in the inferior nasal quadrant was made with the parents, with the aim of improving both the IOP control and cosmesis. The surgery was performed similarly to that described above. The tube was intubated with a ripcord of 3–0 non-absorbable monofilament polyamide suture and was ligated with 7–0 vicryl to prevent hypotony post-surgery.

One month postoperatively, the IOP was 35 mmHg in the right eye and was 11 mmHg in the left eye (with three anti-glaucoma drops OU). The patient and the parents were happy with the appearance of the left eye following surgery and opted for a similar procedure in the right eye. The patient subsequently needed trimming of the tube in the right eye and removal of both ripcords.

The patient and her parents were happy with the cosmesis after the exchange in both eyes (**Figure 2B**). The IOP control for the right Baerveldt implant lasted 7 years (with the IOP below 18 mmHg with glaucoma medication) from the exchange until she needed subsequent glaucoma surgery to control the IOP. The left Baerveldt implant lasted only 2 years, with the left eye thereafter needing another tube to control the IOP. The patient’s best correct visual acuity was maintained at 20/100 in the right eye and at 20/400 in the left eye. Her optic nerve cupping was 0.85 in the right eye and was 0.9 in the left eye.

## Discussion

This report describes two patients who had exchange of a valved for a non-valved GDD for glaucoma control and cosmesis. While the exchange has been reported for glaucoma control (1, 3), this is the first report documenting the improvement in cosmesis that occurs after the exchange of a valved inferior GDD.

Zuo and Lesk (1) published the first report of a non-valved/valved GDD exchange. They described nine eyes that had an exchange and reported surgical success in seven out of nine patients, with a 36% mean reduction in IOP. In a more recent report, Jacobson and Bohnsack (3) reported the results on 12 eyes of 10 children who underwent Ahmed/Baerveldt exchange for glaucoma control with a 100% surgical success at 1 year. Although the procedure was considered effective for normalizing IOP, the previous reports did not document the change in cosmesis that occurs after the exchange. In addition to Ahmed valve/Baerveldt GDD, which has previously been described, we report on one patient who had an exchange of an Ahmed valve for an AADI, thus suggesting that the technique of exchange is generalizable for any Ahmed valve/non-valved GDD exchange.

Both of our patients had raised concerns over the appearance of encapsulated inferior valved GDDs. In the first patient, a new GDD could have been implanted in a different quadrant. However, as the patient had voiced concerns over the appearance of the encapsulated valved GDD, exchange was considered a good option to achieve both IOP control and improvement in cosmesis. In contrast, in the second patient, there was extensive conjunctival scarring due to multiple previous surgeries, and further glaucoma surgery in another quadrant was not a viable option. As such, exchange of the GDD was considered a surgical option to achieve IOP control and to improve the poor cosmetic appearance resulting from the encapsulated AGVs.

While the use of valved GDDs is an effective and safe surgical option, these devices are known to have a considerable failure rate in the longer term in younger patients (4) due to encapsulation of the device and the fibrous ingrowth within the valve leaflets (5). The encapsulation can be disfiguring, especially for inferiorly placed GDDs in the presence of scleral show. Cosmetically, this can be undesirable, especially for younger patients. Non-valved devices typically result in a more posterior and diffuse bleb, resulting in a better appearance of the plate and capsule compared with encapsulated valved GDDs.

Although we have reported valved/non-valved exchange for IOP control, there are multiple options for achieving IOP control after a valved GDD failure. These include revision (6), needling of the plate (7), or cycloablation. Revision of valved devices has shown limited short-term success (6), and cyclophotocoagulation is also generally short-lived in children and often needs to be repeated. We believe that a valved/non-valved GDD exchange offers a better chance of long-term IOP control in younger people. Several studies have shown that, in young people, non-valved GDDs achieve better IOP control (8) with less medication than valved devices (9).

While we advocate the exchange of inferior valved for non-valved GDDs for IOP control and cosmesis, it should be borne in mind that this is not a straightforward surgery. Exchange requires careful dissection of the valved GDD from the original quadrant while carefully preserving the conjunctiva, and this can be cumbersome and time-consuming. With repeated tissue dissection and manipulation, there is also a concern about subsequent GDD exposure.

We followed the two patients for more than 7 years after the exchange. To the date of writing this report, the implant in the first patient still provides good IOP control with topical medications. The implants in the second patient provided IOP control for 7 years in the right eye and 2 years in the left eye. The second patient had a long history of multiple failures of all the previous glaucoma surgeries that she had in both eyes at a rate of one glaucoma surgery in each eye for the first 8 years of her life. We believe that we have succeeded in improving the cosmesis in both eyes and the IOP control in the right eye as the IOP was controlled for 7 years. In the left eye, we partially succeeded in controlling the IOP as it was hoped that the exchange would provide the IOP for a time longer than only 2 years. The early failure of the IOP control in the left eye could be due to the advanced disease in this eye, and it was known that it would be more difficult to control the IOP in this eye as it had one more trabeculotomy and one cyclodiode laser ablation compared to the right eye.

Both of the patients in this report indicated that they were satisfied with the postoperative appearance of their eyes. However, we did not conduct a detailed study of postoperative cosmesis or use a formal questionnaire for this purpose. Another obvious limitation of this report is that it was confined to only two patients. A more detailed report on a larger group of patients is needed to confirm the results of this study.

Both of our patients had inferior valved GDDs, and cosmetic improvement was noted in both of them. In general, the first choice for GDDs is the superotemporal quadrant, where the plate is well covered by the eyelid. Therefore, we do not expect that the same cosmetic findings would be generalizable to superior GDDs.

## Conclusions

In summary, we report on two cases of valved/non-valved GDD exchange for IOP control and for improvement of the cosmetic appearance of the eyes. Our preliminary results indicate that this technique could be more widely adopted for patients with encapsulated valved GDDs who have poor IOP control and are bothered about the unsightly appearance of their inferior valved devices.

## Data availability statement

The original contributions presented in the study are included in the article/supplementary material. Further inquiries can be directed to the corresponding author.

## Ethics statement

The studies involving humans were approved by King Khaled Eye Specialist Hospital IRB. The studies were conducted in accordance with the local legislation and institutional requirements. Written informed consent for participation was not required from the participants or the participants’ legal guardians/next of kin in accordance with the national legislation and institutional requirements. Written informed consent was obtained from the participant/patient(s) for the publication of this case report.

## Author contributions

AK: Data curation, Project administration, Writing – original draft. MA: Data curation, Project administration, Writing – original draft. RM: Conceptualization, Supervision, Writing – review & editing.
